# Macropinocytosis regulates cytokine expression through Erk signaling in LPS-stimulated macrophages

**DOI:** 10.1247/csf.25008

**Published:** 2025-03-08

**Authors:** Li Wang, Yanan Li, Yuxin He, Yuchen Fang, Hitomi Mimuro, Adam C. Midgley, Sei Yoshida

**Affiliations:** 1 State Key Laboratory of Medicinal Chemical Biology, College of Life Sciences, Frontiers Science Center for Cell Responses, Nankai University, Tianjin, China; 2 Division of Genome-Wide Infectious Microbiology, Research Center for GLOBAL and LOCAL Infectious Diseases, Oita University, Oita, Japan; 3 Key Laboratory of Bioactive Materials, Ministry of Education, College of Life Sciences, Nankai University, Tianjin, China; 4 Nankai International Advanced Research Institute, Shenzhen, China

**Keywords:** macropinocytosis, Erk, IL-1β, IL-10

## Abstract

Macropinocytosis, a type of large-scale endocytosis process, is induced in macrophages by extracellular stimuli, including lipopolysaccharide (LPS). In addition to uptake function, emerging evidence supports a link between macropinocytosis and LPS-induced signal transduction. Following LPS stimulation, membrane ruffles are induced to form cup-like structures known as macropinocytic cups, a necessary precursory step for macropinocytosis. We have recently shown that Akt is activated at the cups and is an upstream regulator of the Iκ-B/NF-κB pathway implicated in the production of IL-1α and IL-6. Here, we further investigated the molecular mechanisms and show that the macropinocytic cups also regulated the Ras/Mek/Erk/c-Fos pathway to modulate IL-1β expression independently of the Akt pathway. In addition, we observed that the cup-dependent Akt pathway downregulated the expression of IL-10, in which the activation of the Erk pathway was critical. Taken together, we propose that macropinocytic cups separately modulate the Akt and Erk pathways in cytokine expression.

## Graphical Abstract

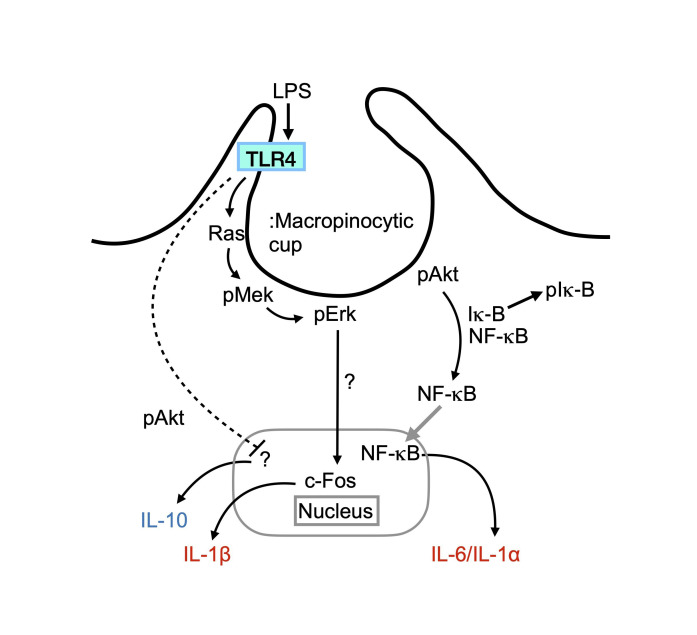

## Introduction

Macropinocytosis is a large-scale endocytosis process, which can be induced in response to extracellular stimuli, including growth factors, cytokines, chemokines, and lipopolysaccharide (LPS). The macropinocytosis process is predominantly observed in phagocytes such as macrophages and dendritic cells and functions as a mechanism to ingest bacteria, cell debris, and extracellular nutrients (Doodnauth *et al.*, 2019; Lin *et al.*, 2020; Stow *et al.*, 2020). The morphological patterns and molecular mechanisms of macropinocytosis in macrophages have been well studied, mainly using imaging techniques (Salloum *et al.*, 2023; Swanson, 2008; Swanson and Araki, 2022). Upon ligand stimulation of cells, activation of phosphoinositide 3-kinase (PI3K) and small guanosine triphosphate hydrolases (GTPases) induce the formation of dynamic membrane ruffles, and a proportion of these ruffles form cup-like structures known as macropinocytic cups. Macropinocytic cups envelop extracellular spaces to generate macropinosomes, large vesicles that move toward the center of the cytosol and fuse with lysosomes (Doodnauth *et al.*, 2019; Lin *et al.*, 2020; Stow *et al.*, 2020; Swanson, 2008).

In addition to the ingestion roles, macropinocytosis has been shown to be involved in signal transduction mechanisms in various cell types, such as in mouse embryonic fibroblasts (MEFs) (Palm *et al.*, 2015; Yoshida *et al.*, 2015), podocytes (Hua *et al.*, 2023), cancer cells (Commisso *et al.*, 2013; Sun *et al.*, 2022; Zdzalik-Bielecka *et al.*, 2021), and macrophages (Lucas *et al.*, 2021; Pacitto *et al.*, 2017; Wang *et al.*, 2024). AKT is one of the canonical signaling molecules that regulates cell growth and differentiation (Manning and Toker, 2017; Sugiyama *et al.*, 2019). AKT and kinase molecules possess pleckstrin homology (PH)-domains that interact with Phosphatidylinositol (3,4,5)-trisphosphate (PIP3), a product of PI3K (Fruman *et al.*, 1999; Lemmon, 2007). Therefore, when PI3K is activated after extracellular stimulation, PIP3 is generated in the membrane, where these molecules gather, phosphorylating AKT and activating the pathway. Indeed, using imaging and biochemical analysis, we have previously shown that PIP3 accumulated at macropinocytic cups, recruiting AKT and other kinases to the cup regions, whereas inhibiting cup structure formation attenuated the phosphorylation of AKT (Wang *et al.*, 2024; Yoshida *et al.*, 2018). These observations indicated that macropinocytic cups function as signaling platforms of the AKT pathway (Swanson and Yoshida, 2019).

In our recent research, we uncovered the role of cup-dependent Akt signaling in driving macrophage cytokine production using mouse cell lines and primary mouse macrophages (Wang *et al.*, 2024). Other work has also shown that AKT has important roles in regulating transcription factors that in-turn modulate cytokine expression in macrophages (Manning and Toker, 2017; Vergadi *et al.*, 2017). NF-κB is one of the main transcription factors that becomes activated after LPS stimulation and modulates cytokine expression (Yu *et al.*, 2020). We found that the Iκ-B/NF-κB pathway was activated downstream of cup-dependent Akt phosphorylation in LPS-stimulated mouse macrophages, and inhibition of macropinocytic cup formation attenuated the expression of the pro-inflammatory interleukins, IL-1α and IL-6 (Wang *et al.*, 2024). Based on these findings, we proposed that macropinocytic cups modulate LPS-stimulated cytokine expression via the Akt/Iκ-B/NF-κB pathway. Using mouse macrophage cell lines and primary mouse macrophages, we observed that the inhibition of cups, but not the Akt pathway, blocked the expression of IL-1β. In addition, inhibition of cups increased the expression of the anti-inflammatory interleukin, IL-10. These observations suggested that there are additional functions that can be attributed to macropinocytosis in the context of cytokine expression and their regulatory mechanisms (Wang *et al.*, 2024).

Similar to the AKT pathway, the MEK/ERK pathway has been shown to regulate cytokine expression (Carter *et al.*, 1999; Stierschneider and Wiesner, 2023; Tsuchiya *et al.*, 2015). Following LPS stimulation, small GTPase Ras is activated as an upstream regulator of MEK, resulting in ERK activation (Ain *et al.*, 2020; Guha *et al.*, 2001; Lim *et al.*, 2017; Wu *et al.*, 2020). In-turn, the transcription factor c-Fos can be activated downstream of ERK (Seo *et al.*, 2018; Wu *et al.*, 2020; Yang *et al.*, 2016). Through interaction with the c-Jun transcription factor, c-Fos and c-Jun form Activator Protein-1 (AP-1) (Song *et al.*, 2023). It has been suggested the AP-1 regulation by the ERK pathway is implicated in LPS-stimulated pro-inflammatory cytokine expression (Seo *et al.*, 2018; Yang *et al.*, 2016). Interestingly, we observed that Ras and Erk were spatially located at the LPS-induced macropinocytic cups and that inhibition of cup formation blocked LPS-stimulated Erk phosphorylation in mouse macrophages (Wang *et al.*, 2024). Therefore, we hypothesized that macropinocytic cups regulate the Erk pathway as a part of the mechanism that drives IL-1β and IL-10 expression.

In the present study, we investigated the role of macropinocytosis in the LPS-stimulated Ras/Mek/Erk/c-Fos signaling pathway. Our results showed that both Mek and Erk were phosphorylated at the cups and that inhibition of cups blocked the Mek/Erk signaling pathway. Inhibition of cups and the Erk pathway attenuated the nuclear localization of c-Fos. Gene expression analysis showed that inhibition of cups and the Erk pathway, but not the Akt pathway, decreased the expression of LPS-induced IL-1β. Interestingly, gene expression analysis also revealed that the inhibition of cups or the Akt pathway increased IL-10 expression whereas inhibition of Erk pathway decreased IL-10 expression. Taken together, our results suggest that macropinocytic cups serve as the initiating platforms that are important in driving the expression of inflammation-associated cytokines in macrophages through the separate activation of the Akt/Iκ-B/NF-κB and Mek/Erk/c-Fos pathways.

## Materials and Methods

### Cells, reagents, and antibodies

Macrophage cell line RAW264.7 and the mouse podocyte cell line MPC5 were purchased from Tongpai Biotechnology (Shanghai, China). HeLa, 293T, and human hepatocellular carcinoma cell line Huh7 were purchased from Hunan Fenghui Biotechnology (Changsha, Hunan, China). Bone marrow derived macrophages (BMMs) were prepared as described previously (Wang *et al.*, 2024; Yoshida *et al.*, 2009; Yoshida *et al.*, 2015). The RAW264.7 cells and BMMs were cultured in the Roswell Park Memorial Institute (RPMI) 1640 medium (Gibco, C11875500BT) or Dulbecco’s modified Eagle Medium (DMEM) (Gibco, C11995500BT) respectively, each supplemented with 10% fetal bovine serum (FBS) (Excell, Suzhou, China, FND500), penicillin (B25911; Shanghai Yuanye Bio-Technology), and streptomycin (A610494-0050; Sangon Biotech, Shanghai, China). All other cells were cultured in DMEM supplemented with 10% FBS, penicillin and streptomycin. LPS (L6511-10MG) was purchased from Sigma–Aldrich (St. Louis, USA). For Western blotting and qPCR analysis, we cultured 1–2 × 10^6^ cells (or until 80% confluency) in one well of 6-well plates (TCP010006; Jet Bio-Filtration, Guangzhou, China) as one sample. 5-(*N*-Ethyl-*N*-isopropyl)-amiloride (EIPA) (1154-25-2) and MK2206 (M1837) were purchased from Tocris Bioscience (Bristol, UK) and AbMole Bioscience (Houston, TX, USA), respectively. Wiskostatin (#ab141085) was purchased from Abcam (Boston, USA). U0126 (#9903) was purchased Cell Signaling Technology (Danvers, USA). Anti-ERK1/2 (#4695) and anti-pERK1/2 (#4376) antibodies were from Cell Signaling Technology. Anti-MEK1/2 (11049-1-AP), anti-LAMP1 (CD107a) (65050-1-lg), and anti-lamin A/C (10298-1-AP) antibodies were from Proteintech (Wuhan, China). Anti-pMEK1/2 antibody (#AF8035) was from Affinity (Changzhou, China). Anti-GSK3β (A11731), anti-pGSK3β (AP0039), anti-TLR4 (A0007), and anti-Ras (A19779) antibodies were purchased from ABclonal (Wuhan, China). Anti-c-Fos antibody (#340249) was purchased from Zenbio (Chengdu, China). Rhodamine phalloidin (RM02835) was purchased from ABclonal. Mounting medium with DAPI (ab104139) was purchased from Abcam.

### Immunofluorescence (IF) staining and confocal microscopy

For IF staining, we cultured the cells (until 80% confluency) on a coverslip (BS-14-RC; Bio Sharp, Hefei, China) in one well of 24-well plates (TCP010024; Jet Bio-Filtration, Guangzhou, China) as one sample. After each assay, the staining was performed as described previously (Sun *et al.*, 2022; Wang *et al.*, 2024). Briefly, cells were fixed in 4% paraformaldehyde (PFA; SparkJade, Qingdao, China, EE0001) for 30 min at room temperature (RT), then permeabilized with 0.1% TritonX-100 for 5 min before blocking in 5% BSA for 30 min. The cells were incubated with the indicated primary antibodies overnight at 4°C, washed with Tris-buffered saline containing Tween-20 (TBST) for 5 minutes three times, and stained with secondary antibodies, anti-rabbit IgG Alexa Fluor 488 (Abcam 150081) or anti-rat IgG Dylight 488 (Abbkine, Georgia, USA, A23240). Cell images were captured with a TCS SP5 Leica confocal microscope at the Core Facility of the College of Life Science at Nankai University. ImageJ software was used for the line-scan analysis of the obtained confocal images. The macropinocytic cup assay was performed as previously described (Wang *et al.*, 2024). Briefly, after fixation and staining, DAPI images were used to count the number of observed cells, while actin labelled cell images were used to identify and count the cup structures. More than 10 images from two independent experiments were performed to calculate the average using “number of cups”/“number of observed cells.”

### Inhibitor treatment, preparation of cell lysate, and western blot analysis

For the inhibitor treatment, the cells were pretreated with EIPA (25 μM) or MK2206 (2 μM) or U0126 (10 μM) for 30 min or with wiskostatin (10 μM) for 20 min, before LPS stimulation (15 min, 1 μg/mL). For western blot analysis, cells were lysed in CHAPS buffer (40 mM HEPES pH 7.5, 120 mM NaCl, 1 mM EDTA, 10 mM pyrophosphate, 10 mM glycerophosphate, 1.5 mM Na_3_VO_4_, 0.3% CHAPS) supplemented with protease inhibitors (Roche, 04693159001) or buffers provided with the Nuclear and Cytoplasmic Protein Extraction Kit (Beyotime, Shanghai, China, P0028) as previously described (Wang *et al.*, 2024). The isolated protein samples were assessed by Western blot, as previously described (Wang *et al.*, 2024).

### Quantitative real-time PCR (qPCR)

Total RNA was extracted from cells using the Eastep Super Total RNA Extraction Kit, following the manufacturer’s instructions (Promega, Madsion, USA, LS1040). Then total RNA was reverse transcribed to cDNA according to the kit manufacturer’s protocols (*Evo M-MLV* RTPremix kit Accurate Biology, Changsha, China, AG11706). The qPCR was performed using the 2x RealStar Power SYBR Green qPCR mix kit (Genstar, Beijing, China, A311) and a Light Cycle 96 machine (Bio-Rad, 1855202). The primers were as follows:

IL-1β-F: 5'-CAACCAACAAGTGATATTCTCCATG-3'

IL-1β-R: 5'-GATCCACACTCTCCAGCTGCA-3'

IL-10-F: 5'-GGTTGCCAAGCCTTATCGGA-3'

IL-10-R: 5'-ACCTGCTCCACTGCCTTGCT-3'

HPRT-F: 5'-GCAGTACAGCCCCAAAATGG-3'

HPRT-R: 5'-AACAAAGTCTGGCCTGTATCCAA-3'

### Quantification and statistical analysis

GraphPad software (version 8.0) was used for the quantification and statistical analysis. Details of each quantification and statistical analysis used are provided in the Figure legends.

## Results

### Ras/Mek/Erk pathway is activated at macropinocytic cups regions in LPS-stimulated macrophages

**Fig. 1:**
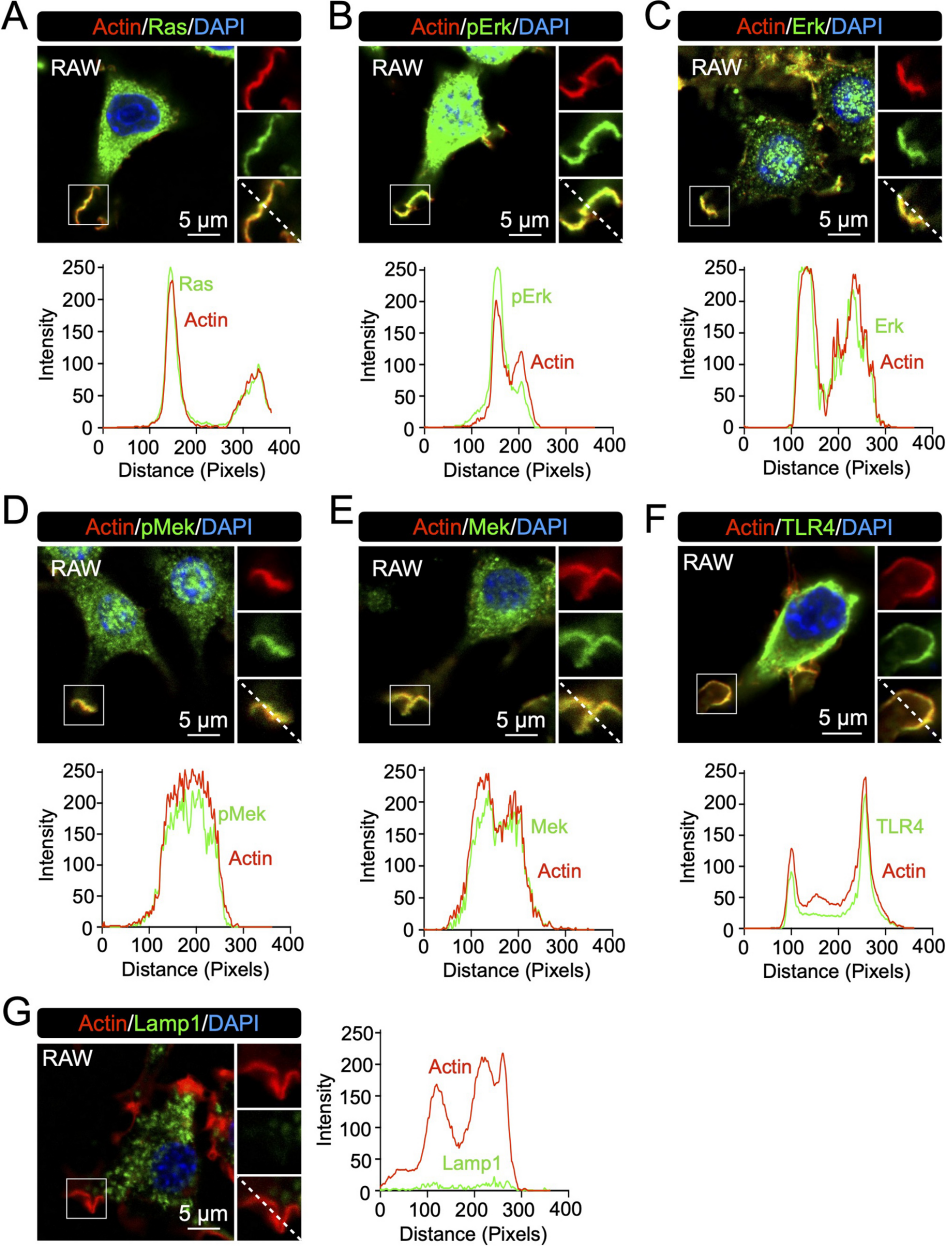
Confocal images showing that the Ras/Mek/Erk pathway is activated at LPS-induced macropinocytic cups Representative confocal images of Actin/Ras (A), Actin/pErk (B), Actin/Erk (C), Actin/pMek (D), Actin/Mek (E), Actin/TLR4 (F), and Actin/Lamp1 (G), in RAW264.7 cells are shown. Results of line-scan analysis according to dot lines in the enlarged marge images are shown as graphs. Lamp1 was observed as a negative control.

**Fig. 2:**
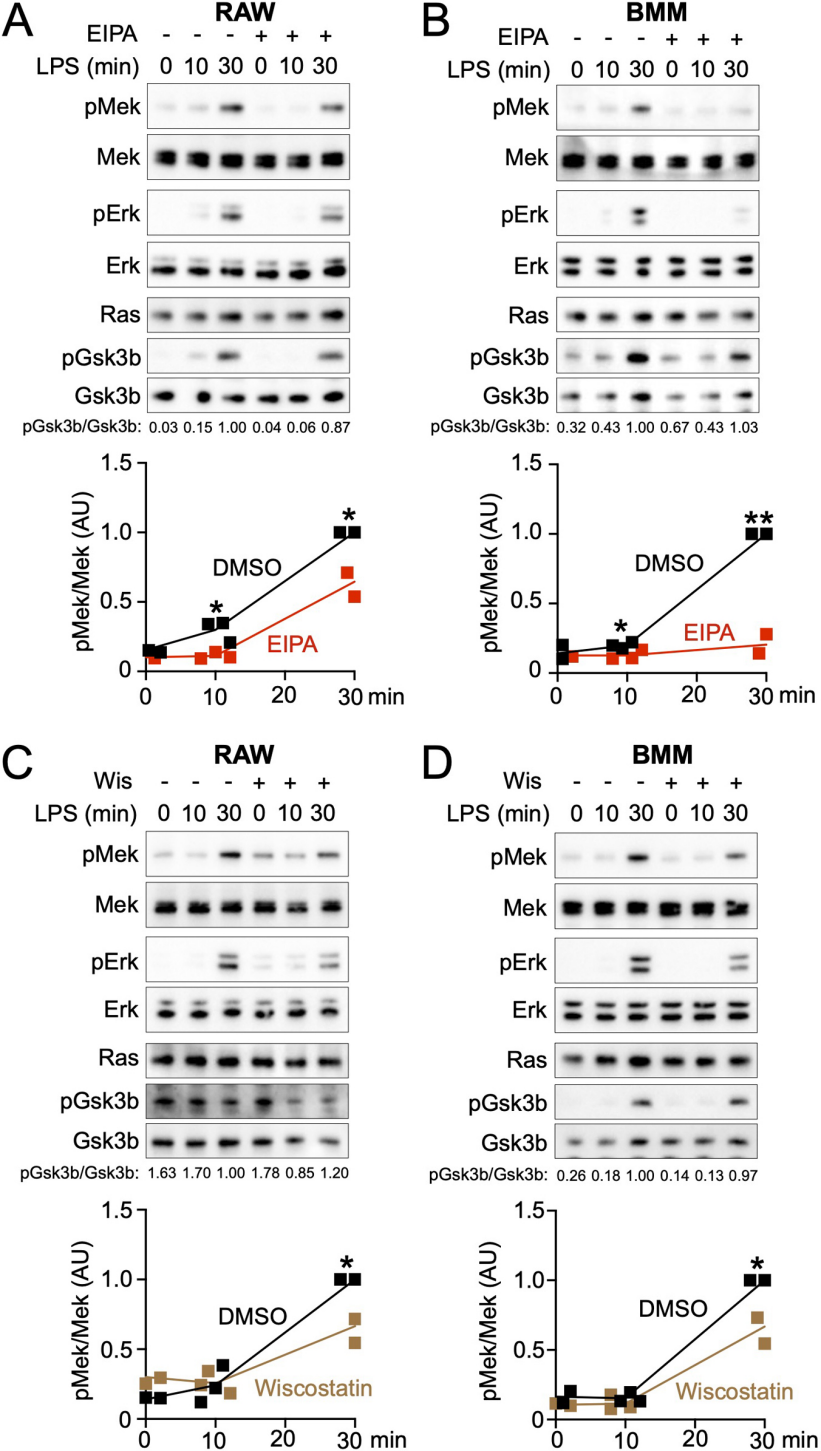
Western blot showing that inhibition of cups blocks pMEK (A and B) EIPA treatment blocked LPS-induced pErk and pMek in RAW264.7 cells (A) and BMMs (B). (C and D) Wiskostatin treatment blocked LPS-induced pErk and pMek in RAW264.7 cells (C) and BMMs (D). Ras was used as a control to confirm the protein amount applied for the analysis was the same in each sample. Ratio value of pGsk3b/Gsk3b are shown at the bottom of the images, indicating that cells after EIPA treatment were still viable. Results of quantification analysis from three independent experiments are shown as graphs *P<0.05, **P<0.01, one-tailed Student’s *t*-test. Results are presented as arbitrary units (AU). Original WB images of these data are shown as Supplemental Materials.

As we observed in our previous study (Wang *et al.*, 2024), both Ras and Erk were confirmed to be located at regions coinciding with formed LPS-induced macropinocytic cups in RAW264.7 cells, and that Erk was phosphorylated (Fig. 1A–C, Supple-Fig. 1A–C). These observations suggest that Mek, which is downstream of Ras and upstream of Erk, is also activated at the cups. Indeed, confocal microscopy and the line-scan analysis showed there was prominent Mek and phosphorylated Mek (pMek) signals that coincided with cup regions (Fig. 1D and E, Supple-Fig. 1D and E). We also observed that toll-like receptor 4 (TLR4) accumulated at the location of cups, although not exclusively as signal could also be identified elsewhere (Fig. 1F, Supple-Fig. 1F). In our previous work, we used Lamp1 signal as a negative control for the observation of cup-dependent signals (Wang *et al.*, 2024). Line-scan analysis confirmed that the protein was not located at the cups (Fig. 1G, Supple-Fig. 1G). We repeated these observations in BMMs, and detected Mek, pMek, and TLR4, but not Lamp1, at the cups (Supple-Fig. 2). These results suggested that the Ras/Mek/Erk pathway is activated at the cups as a part of LPS-stimulated and TLR4-mediated signal transductions.

### Inhibition of macropinocytic cups attenuated LPS-stimulated pMek in macrophages

**Fig. 3:**
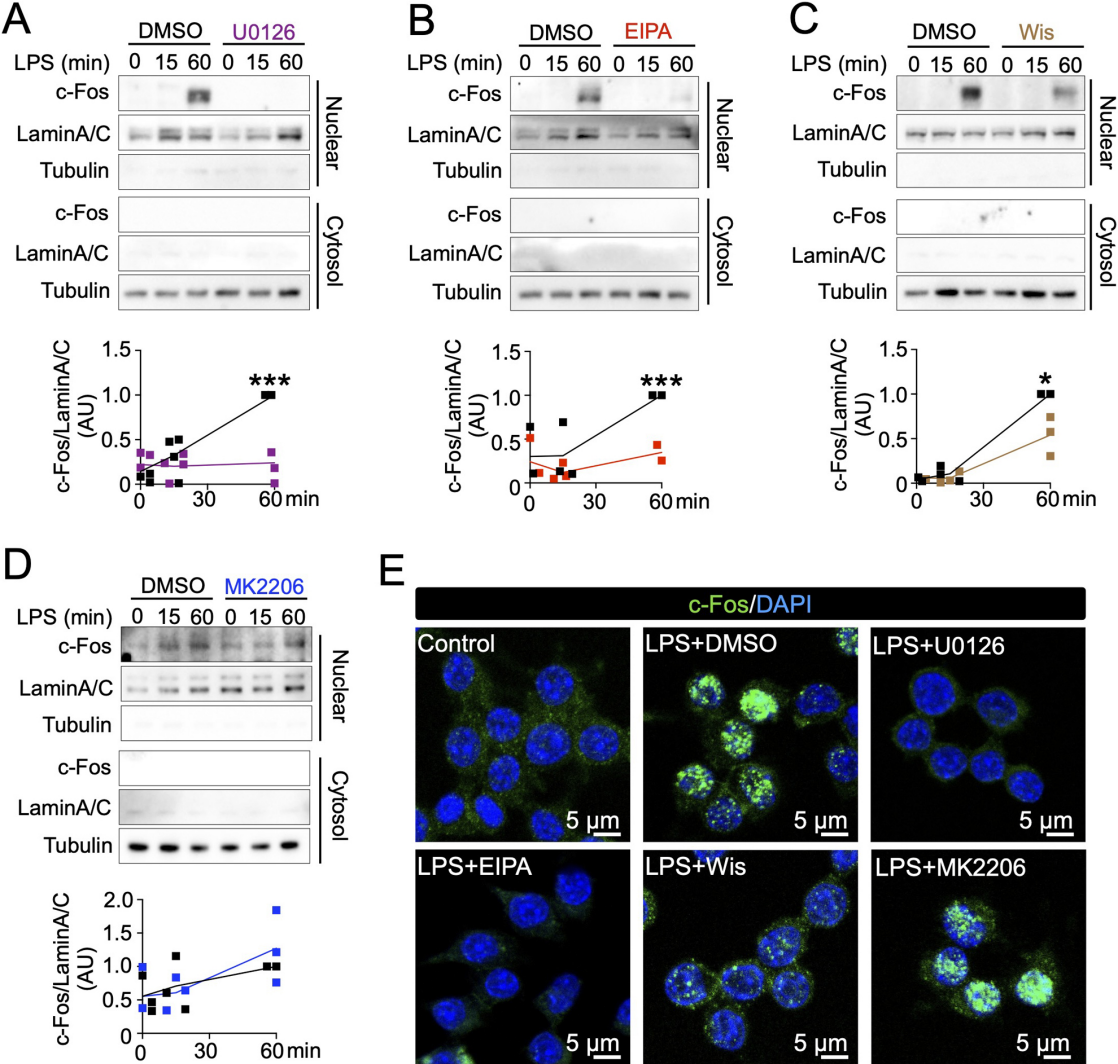
Inhibition of macropinocytic cups block expression of c-Fos in the nucleus (A–D) Treatments of U0126, EIPA, and Wiskostatin, but not MK2206, blocked LPS-induced expression of c-Fos in the nucleus. RAW264.7 cells without/with U0126 (A), EIPA (B), Wiskostatin (C), or MK2206 (D) were stimulated by LPS for 0, 15, and 60 min. Cell lysates were prepared and separated into nuclear and cytosol fractions, which were identified using Lamin A/C and tubulin as the control proteins, respectively. Results of quantification analysis from three independent experiments are shown as graphs. *P<0.05, ***P<0.001, two-tailed Student’s *t*-test. Results are presented as arbitrary units (AU). To confirm the efficacy of the western blot results, see the original WB images shown as Supplementary Materials. (E) Representative confocal images showing that Treatments of U0126, EIPA, and Wiskostatin, but not MK2206, blocked LPS-induced expression of c-Fos in the nucleus.

To examine whether the cup structures are required for LPS-induced Mek activation, we tested the effects of the macropinocytosis inhibitor EIPA and the N-WASP inhibitor Wiskostatin on the Mek/Erk pathway by performing Western blot analysis, as we have previously demonstrated that these inhibitors attenuate LPS-induced macropinocytic cup formation (Wang *et al.*, 2024). We observed that LPS treatment induced pMek at 30 mins in both RAW264.7 cells (Fig. 2A) and BMMs (Fig. 2B). EIPA treatment partially blocked LPS-induced pMek as well as phosphorylated Erk (pErk), and quantification analysis from three independent experiments confirmed these results (Fig. 2A and B). As the control signaling molecules, we also detected Ras, Gsk3b, and phosphorylated Gsk3b (pGsk3b). The ratio value of pGsk3b/Gsk3b during the LPS stimulation was almost the same between the with or without EIPA treatment groups, suggesting that cells remained viable after the inhibitor treatment (Fig. 2A and B). Likewise, pMek and pErk, but not pGsk3B, were attenuated after Wiskostatin treatment (Fig. 2C and D). Additionally, we confirmed the reported data from our previous research, both EIPA and Wiskostatin treatments attenuated LPS-induced pAkt (phosphorylated Akt) (Supple-Fig. 3A–D). Thus, these data suggest that macropinocytic cups are involved in both the LPS-induced Mek/Erk and Akt pathways. It is noted that Mek1/2 from macrophage samples was detected as double bands or a broad band (Supple-Fig. 3E).

### Inhibition of macropinocytic cups disrupts c-Fos function

**Fig. 4:**
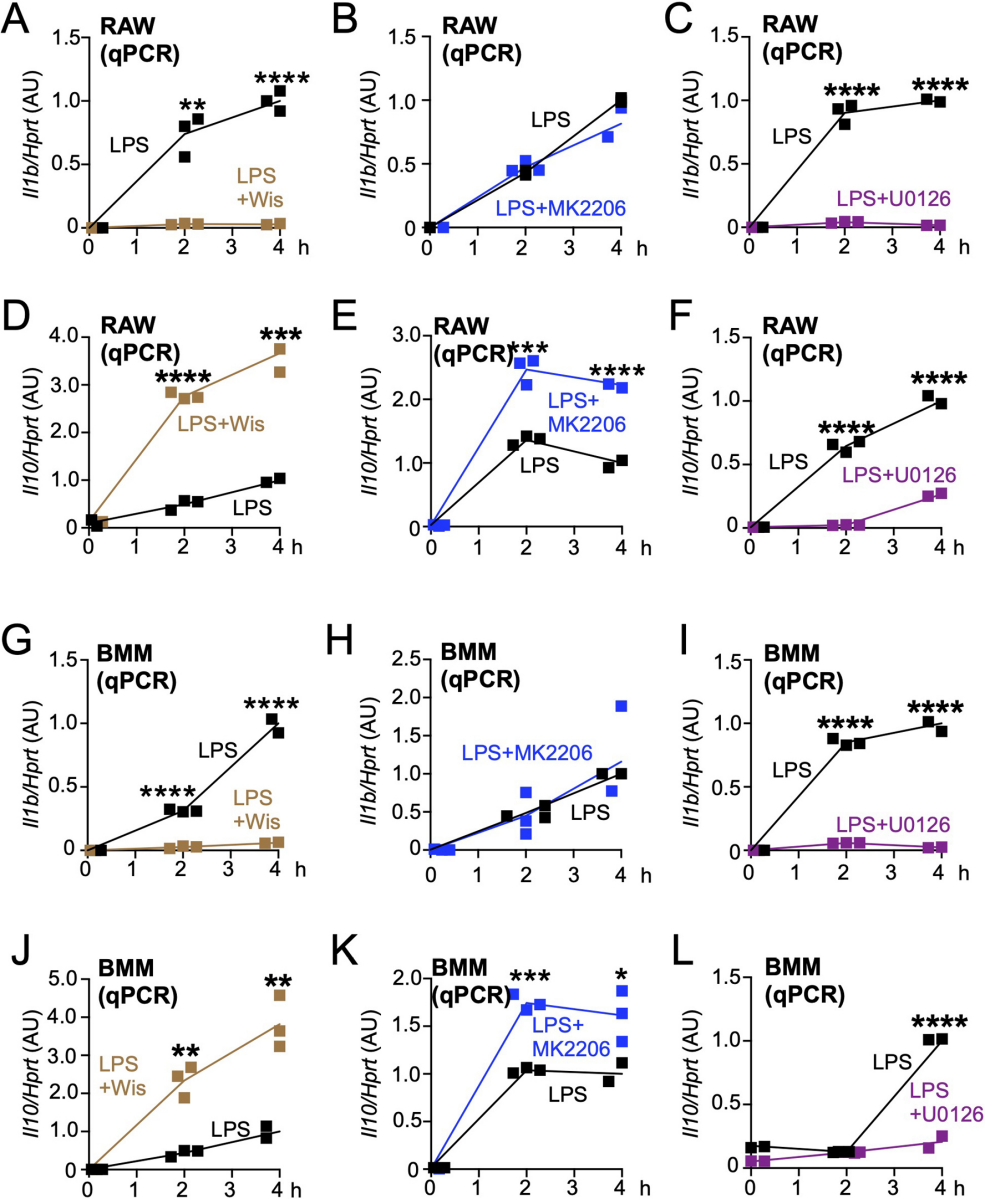
U0126 treatment attenuates LPS-induced IL-1β and IL-10 expression (A–F) Comparison of mRNA levels of IL-1β (*Il1b*) (A–C) and IL-10 (*Il10*) (D–F) in the LPS-stimulated RAW264.7 cells with/without inhibitors. The mRNA levels of IL-1β and IL-10 were analyzed by qPCR. The results were normalized relative to HPRT mRNA levels (*Hprt*) and the ratio values at the indicated time points after the LPS stimulation with DMSO as the control (black), with Wiskostatin (brown), with MK2206 (blue), or with U0126 (purple) treatments are shown. *P<0.05, **P<0.01, ***P<0.001, ****P<0.0001, two-tailed Student’s *t*-test. Three independent experiments were carried out, and one representative result is shown. Results are presented as arbitrary units (AU). (G–L) Comparison of mRNA levels of IL-1β (*Il1b*) (G–I) and IL-10 (*Il10*) (J–L) in the LPS-stimulated BMMs with/without inhibitors. Descriptions of each panel (G–L) are the same as (A–F), respectively, except for cells.

The transcription factor c-Fos, is a downstream transcription factor regulated by the MEK/ERK pathway (Wu *et al.*, 2020). Therefore, if our hypothesis is correct, inhibition of macropinocytic cups would also affect c-Fos activity. Biochemical analysis revealed that expression level of c-Fos in nuclei was increased after LPS stimulation at 60 min, and the expression level was reduced by the MEK inhibitor U0126 treatment (Fig. 3A), which did not block macropinocytic cups (Supple-Fig. 4). As expected, both EIPA and Wiskostatin treatments blocked c-Fos expression to the nucleus (Fig. 3B–C). Since we showed that macropinocytosis regulated LPS-induced Akt (Wang *et al.*, 2024) and it was suggested that Akt activates c-Fos (Jiang *et al.*, 2020), we tested whether inhibition of Akt affected LPS-stimulated c-Fos expression. However, the AKT inhibitor MK2206 treatment did not affect LPS-stimulated c-Fos expression (Fig. 3D). Thus, these results using biochemical analysis suggested that the macropinocytic cups are upstream regulators of the Mek/Erk/c-Fos pathway. To confirm this, we also performed imaging analysis. Confocal microscopy identified strong c-Fos signal in nuclei after LPS treatment at 60 min, and it was observed that treatments with U0126, EIPA, and Wiskostatin, but not MK2206, blocked c-Fos expression in nuclei (Fig. 3E). These results suggest that the cup-dependent Mek/Erk pathway regulates LPS-induced c-Fos activation.

### Macropinocytic cups separately regulate IL-1β and IL-10 expression by modulating the Akt and Erk pathways in LPS-stimulated macrophages

Lastly, we tested the role of the cup-dependent Mek/Erk pathway in cytokine expression by performing qPCR analysis. Confirming our previous work (Wang *et al.*, 2024), Wiskostatin, but not MK2206, treatment blocked LPS-induced IL-1β in RAW264.7 cells (Fig. 4A and B), suggesting that the expression was cup-dependent but Akt-independent. We found that U0126 treatment blocked IL-1β expression (Fig. 4C). Meanwhile, LPS-induced IL-10 was increased after Wiskostatin and MK2206 treatments (Fig. 4D and E), supporting our previous work (Wang *et al.*, 2024). Interestingly, U0126 treatment completely blocked the expression (Fig. 4F). We confirmed that these results were repeatable in BMMs (Fig. 4G–L). Therefore, these new data suggest that macropinocytosis is implicated in LPS-induced cytokine expression, through the orchestration of two separate signal transduction pathways.

## Discussion

**Fig. 5:**
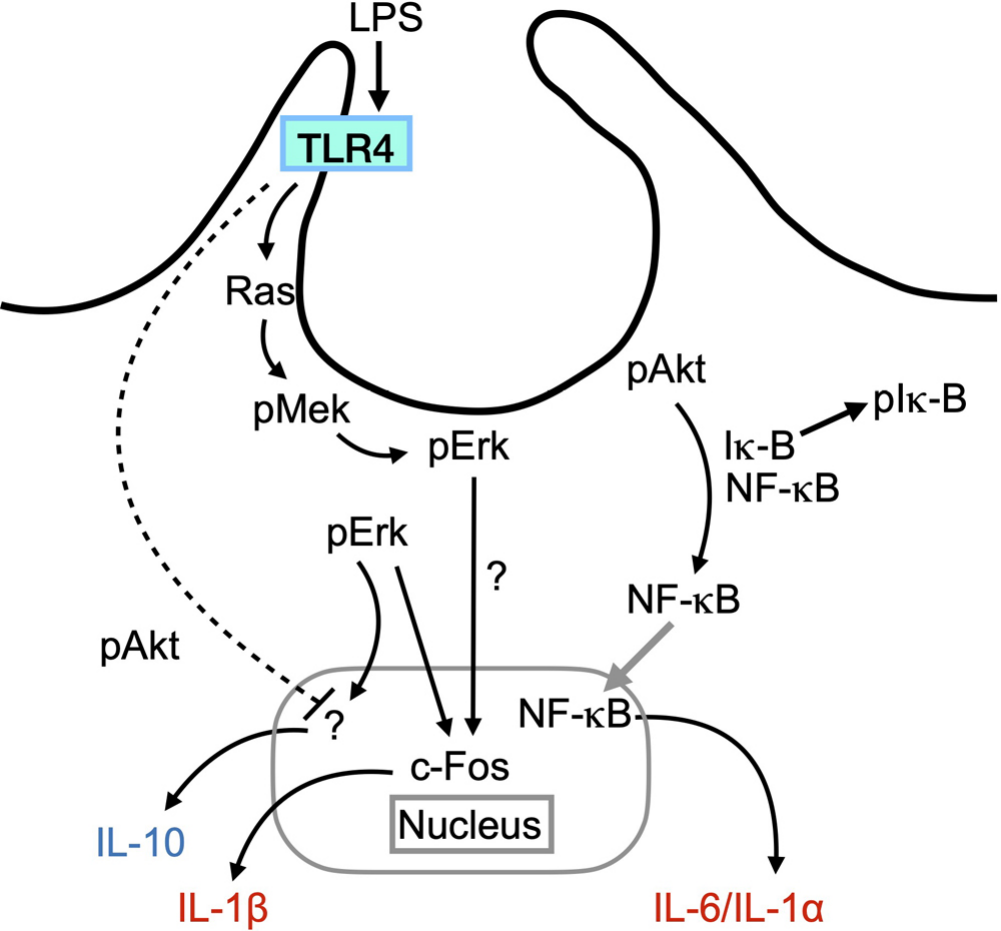
Proposed model of the role of macropinocytic cups in the LPS-induced cytokine expression After LPS stimulation, the Ras/Mek/Erk signaling pathway is activated at macropinocytic cups as a downstream of TLR4. The resulting pErk activates c-Fos in the nucleus, leading to the expression of IL-1β. Meanwhile, the cup separately activates Akt. The resulting pAkt induce phosphorylation of Iκ-B (pIκ-B), which interacts with NF-κB in the cytosol. pIκ-B dissociate the protein complex with NF-κB. NF-κB translocates to the nucleus and initiates the transcription of IL-6 and IL-1α mRNA. Thus, cups separately and cooperatively modulate pro-inflammatory cytokine expression through two distinct pathways. As a third mechanistic insight, it can be suggested that the cup downregulates anti-inflammatory cytokine IL-10 via Akt pathway, while Erk positively regulates the IL-10 expression. The molecular mechanism of c-Fos activation through the cup-dependent Erk pathway and the transcription factor involved in the IL-10 expression remain unknown. In contrast, LPS stimulation induces pErk independent of the cup. This would also lead to c-Fos activation.

This report serves to be complementary to our previous study (Wang *et al.*, 2024). Here, we propose a model that the cup-dependent Akt/Iκ-B/NF-κB pathway positively regulates the pro-inflammatory cytokines IL-6/IL-1α, while the cup-dependent Ras/Mek/Erk/c-Fos pathway regulates the pro-inflammatory cytokine IL-1β (Fig. 5). Therefore, our data suggest that macropinocytic cups separately regulate the Akt/Iκ-B/NF-κB and Ras/Mek/Erk/c-Fos pathways to modulate the expression of IL6/IL-1α and IL-1β, respectively (Fig. 5). Moreover, we observed that the inhibition of cups and the Akt pathway increased the expression of the anti-inflammatory cytokine IL-10, whereas inhibition of the Erk pathway attenuated this expression (Fig 4D–F and J–L). Therefore, these findings suggest that activation of the cup-dependent Akt pathway is implicated in the downregulation of IL-10 expression, in the presence of cup-independent Erk pathway is critical (Fig. 5). Meanwhile, we observed that inhibition of cup formation did not completely block LPS-induced pErk (Fig. 2), indicating the existence of the cup-independent Erk pathway, which would also regulate c-Fos (Fig. 5). Thus, the important role of the cup-dependent Erk pathway should be clarified in the future study.

The molecular mechanism through which Erk in macropinosomes activates c-Fos and the subsequent expression of c-Fos in the nucleus remains to be investigated. Regarding a cellular trafficking mechanism of Erk, recent studies have shown that Erk is recruited by SCIMP (SLP adaptor and CSK-Interacting Membrane Protein), which interacted with TLR4 at the cups (Lucas *et al.*, 2021). The authors also showed that the resulting macropinosomes contained TLR4-SCIMP-Erk complexes, which could activate c-Fos and NF-κB to drive the production of cytokines including IL-1β and IL-6, but not IL-10 (Lucas *et al.*, 2021). It would be proposed that after LPS stimulation, 1) macropinocytic cups are induced, 2) Erk is phosphorylated on the cups (and elsewhere), 3) cups are closed to generate macropinosomes, 4) pErk signaling on the cups is transferred to the resulting macropinosomes, and 5) pErk on the macropinosomes is delivered to c-Fos. Indeed, our data showed that inhibition of macropinocytosis weakly attenuated LPS-induced pErk (Fig. 2), but completely blocked c-Fos activation (Fig. 3) and IL-1β expression (Fig. 4). Therefore, pErk on the cups would be critical for the c-Fos-related IL-1β expression.

Interestingly, it has been shown that LPS-induced pAkt as well as pIκ-B was reduced in CRISPR/Cas9-mediated SCIMP knockout RAW264.7 cells (Luo *et al.*, 2020). We showed that inhibition of macropinocytosis attenuated the Akt/Iκ-B/NF-κB pathway (Wang *et al.*, 2024). Thus, focusing on the role of SCIMP in LPS-induced cytokine expression would be appropriate means to identify the molecular mechanism orchestrating the Akt and Erk pathways in LPS-induced macropinocytosis.

In conclusion, our data from the current study taken together with the findings of previous research, including ours, strongly suggest that macropinocytosis plays an important regulatory role in LPS-induced cytokine expression. Given that cytokines secreted by macrophages have critical roles in immune regulation and its balance (Arango Duque and Descoteaux, 2014; Chen *et al.*, 2023), macropinocytosis would serve a pivotal role through its involvement in mechanisms that adjust the pro/anti-inflammatory balance during bacterial infection. Thus, once the detailed molecular mechanism by which macropinocytosis separately regulates the Akt and Erk pathways is identified, macropinocytosis and macrocytic cup formation have the potential to serve as therapeutic targets for the modulation of pathogen-induced immune responses and inflammation.

## Funding

This study was supported by a Frontiers Science Center for Cell Responses Grant from Nankai University (C029205001) for SY, the Shenzhen Science and Technology Program (Grant No. JCYJ20210324120813037) for SY and ACM, Astellas Foundation for Research on Metabolic Disorders (2023A3542) for HM, and the joint research program of the Research Center for GLOBAL and LOCAL Infectious Diseases, Oita University (2025A02) for HM and SY.

## Conflict of Interest

The authors declare that this study was conducted in the absence of any commercial or financial relationships that could be construed as potential conflicts of interest.

## Data Availability Statement

The datasets presented in this study and the original data are available from the corresponding author upon request. All the original western blot images are shown as the Supplemental Material.

## Author Contributions

LW designed and performed the experiments, with the support of YL, YH, and YF. SY conceived the study, designed the experiments, and wrote the manuscript, with the support of HM and ACM.

## Abbreviations

AP-1: activator protein-1
BMM: bone marrow derived macrophage
DMEM: Dulbecco’s modified Eagle Medium
EIPA: 5-(*N*-Ethyl-*N*-isopropyl)-amiloride
FBS: fetal bovine serum
GTPase: guanosine triphosphate hydrolase
IF: immunofluorescent
Iκ-B: iinhibitor of nuclear factor kappa B
IL: interleukin
LPS: lipopolysaccharide
MEF: moue embryonic fibroblast
NF-κB: nuclear factor kappa B
pAkt/pErk/pGsk3b/pIκ-B/pMek: phosphorylated Akt/Erk/Gsk3b/Iκ-B/Mek
PFA: paraformaldehyde
PI3K: phosphoinositide 3-kinase
PIP3: phosphatidylinositol (3,4,5)-trisphosphate
PH: pleckstrin homology
qPCR: quantitative polymerase chain reaction
RPMI: Roswell Park Memorial Institute
RT: room temperature
SCIMP: SLP adaptor and CSK-Interacting Membrane Protein
TBS/TBST: Tris-buffered saline/TBS containing Tween-20
TLR4: Toll-like receptor 4
