# Supplementary material for: Macropinocytosis regulates cytokine expression through Erk signaling in LPS-stimulated macrophages
Source: Cell Struct Funct. 2025 Mar 8;50(1):103–13. doi: 10.1247/csf.25008 (PMC12702681; doi:10.1247/csf.25008)

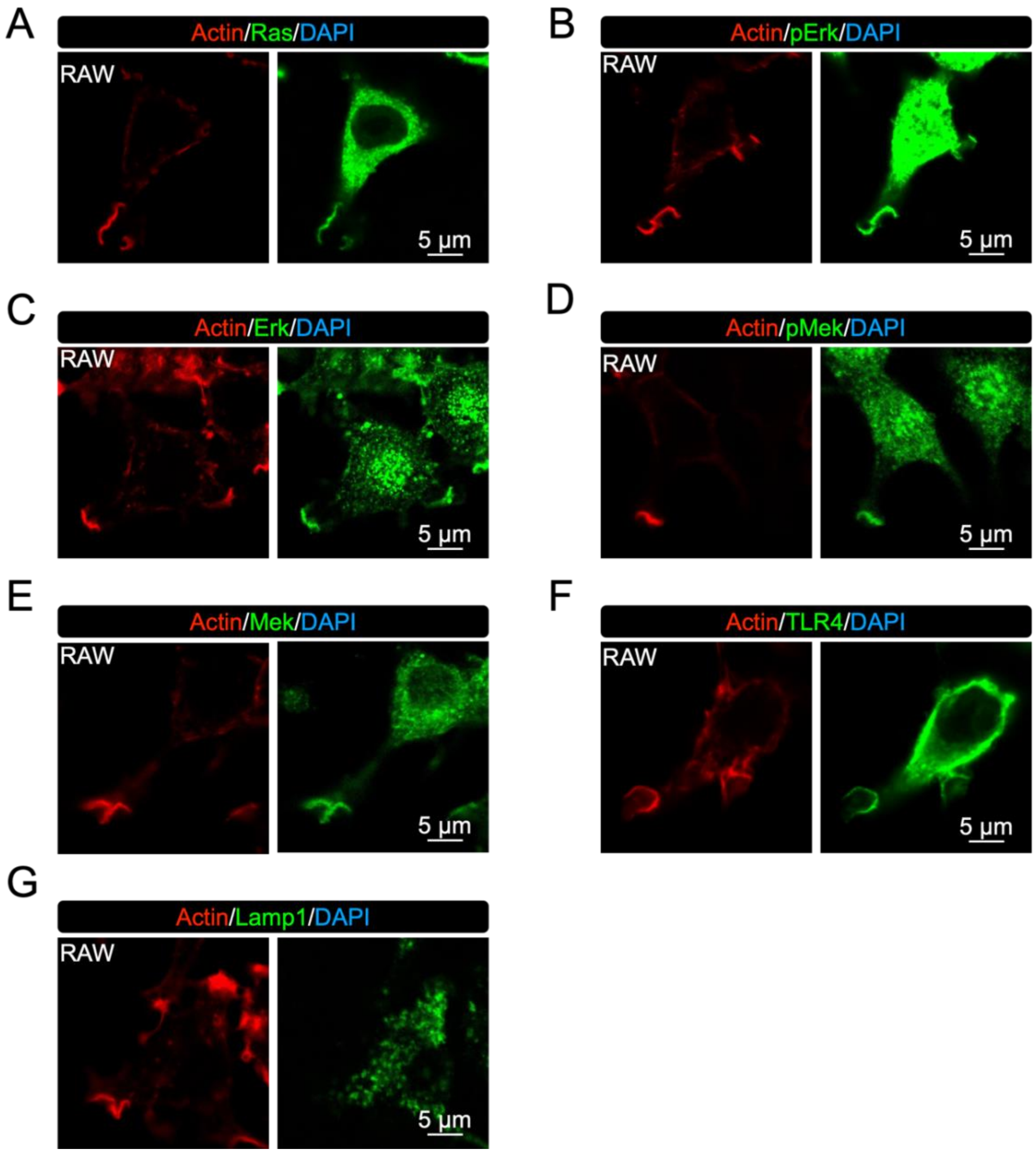

Supplemental-Figure 1. Individual channel images of Figure. 1
Actin/Ras (**A**), Actin/pErk (**B**), Actin/Erk (**C**), Actin/pMek (**D**), Actin/Mek (**E**), Actin/TLR4 (**F**), and Actin/Lamp1 (**G**) are shown. Lamp1 is a negative control.

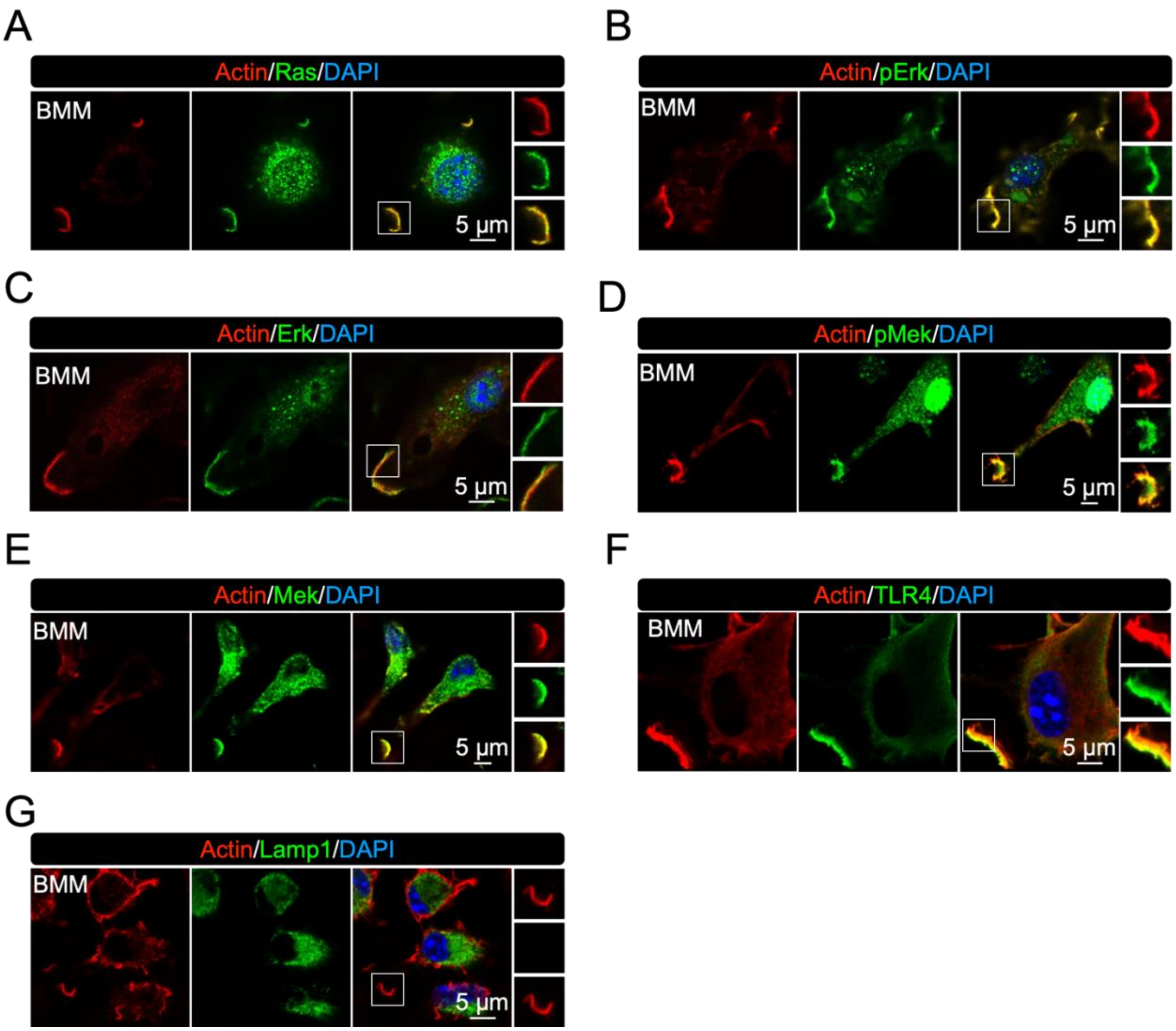

Supplemental-Figure 2. Supplemental confocal images for Figure. 1
Representative confocal images of Actin/Ras (**A**), Actin/pErk (**B**), Actin/Erk (**C**), Actin/pMek (**D**), Actin/Mek (**E**), Actin/TLR4 (**F**), and Actin/Lamp1 (**G**), in BMMs are shown.

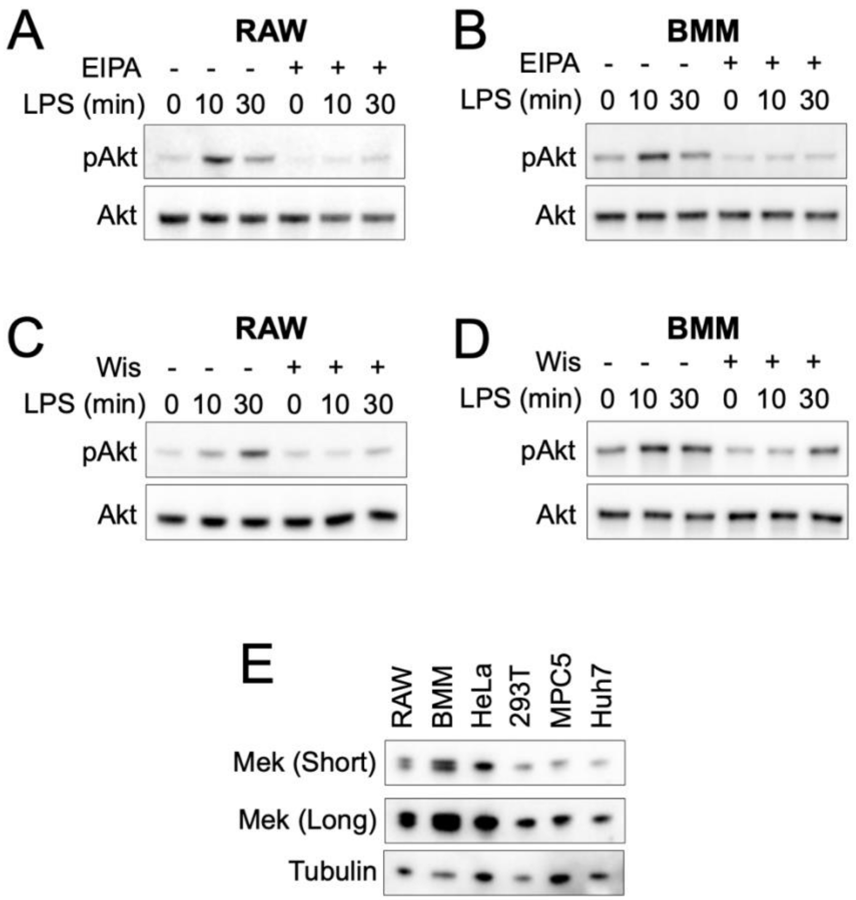

Supplemental-Figure 3. Supplemental data for Figure. 2
(**A-D**) EIPA and Wiskostatin treatments blocked LPS-induced pAkt. (**E**) Comparison of Western blot results of Mek from different cell lines. Mek was detected as double or broad bands in samples from RAW264.7 cells and BMMs. Lysates from HeLa cells, 293T cells, mouse podocyte MPC5 cells, and human hepatocellular carcinoma Huh7 cells were used as controls. Tubulin was detected to adjust the protein expression level between different cell types. Short/Long: Short exposure/Long exposure.

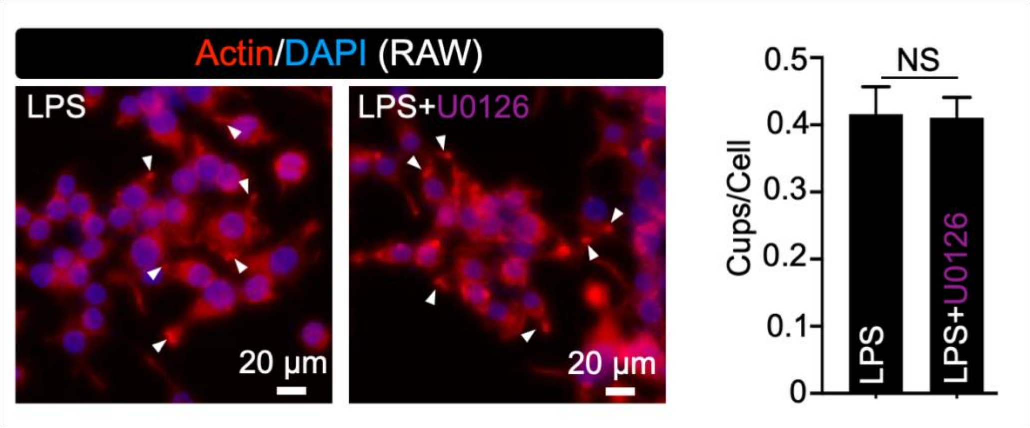

Supplemental-Figure 4. Supplemental data for Figure. 3
U0126 treatment did not block LPS-induced macropinocytic cups. Representative confocal images are shown. Arrows: macropinocytic cups. More than 1000 cells from two independent experiments were counted. NS: not significant. 2-tailed Student's t-test.

Figure 2A

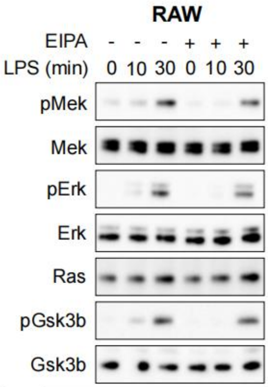

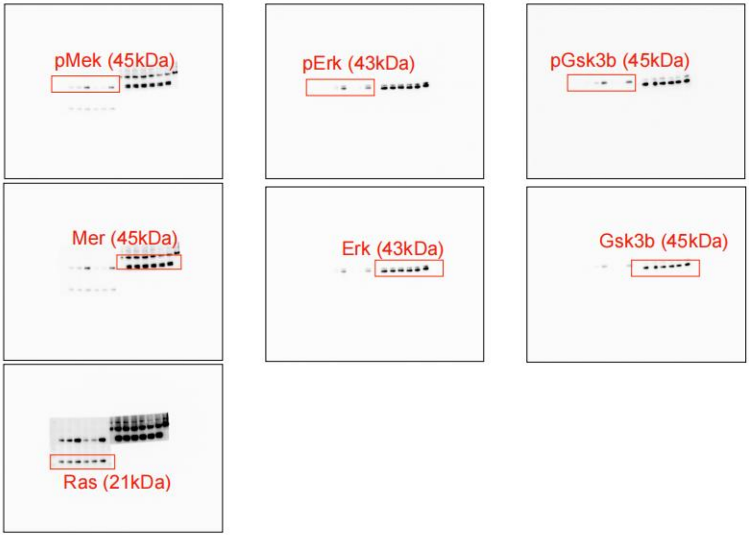

: from the same membrane

pMek | Mek
Ras

: from the same membrane

pErk | Erk

: from the same membrane

pGsk3b | Gsk3b

Figure 2B

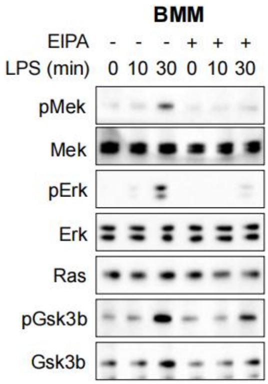

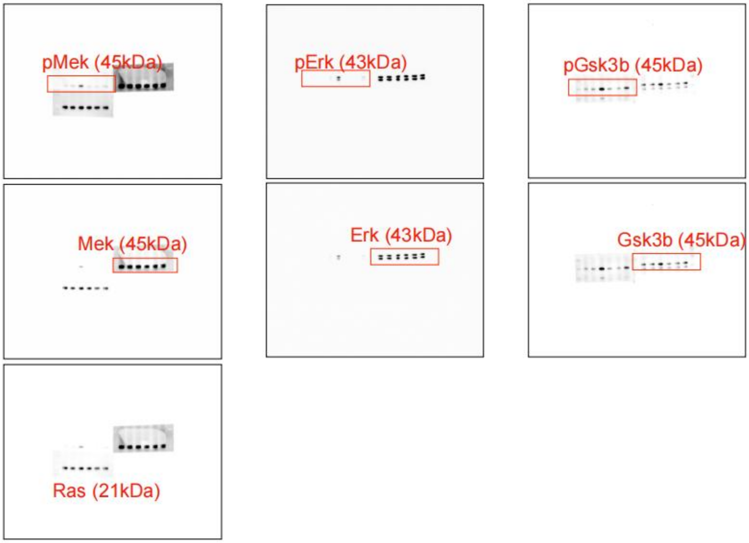

: from the same membrane

pMek	Mek
Ras	

: from the same membrane

pErk | Erk

: from the same membrane

pGsk3b | Gsk3b

Figure 2C

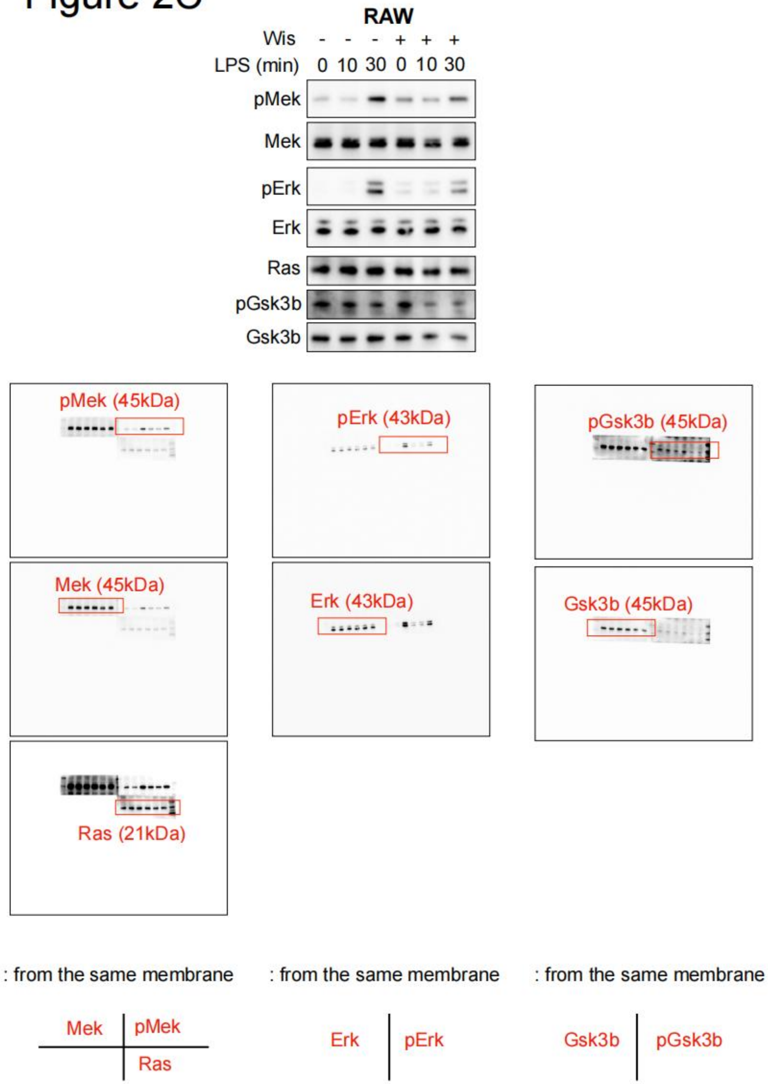

RAW
Wis - - - + + +
LPS (min) 0 10 30 0 10 30
pMek
Mek
pErk
Erk
Ras
pGsk3b
Gsk3b
pMek (45kDa)
pErk (43kDa)
pGsk3b (45kDa)
Mek (45kDa)
Erk (43kDa)
Gsk3b (45kDa)
Ras (21kDa)
: from the same membrane
: from the same membrane
: from the same membrane
Mek
pMek
Ras
Erk
pErk
Gsk3b
pGsk3b

Figure 2D

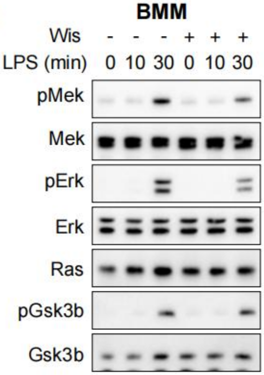

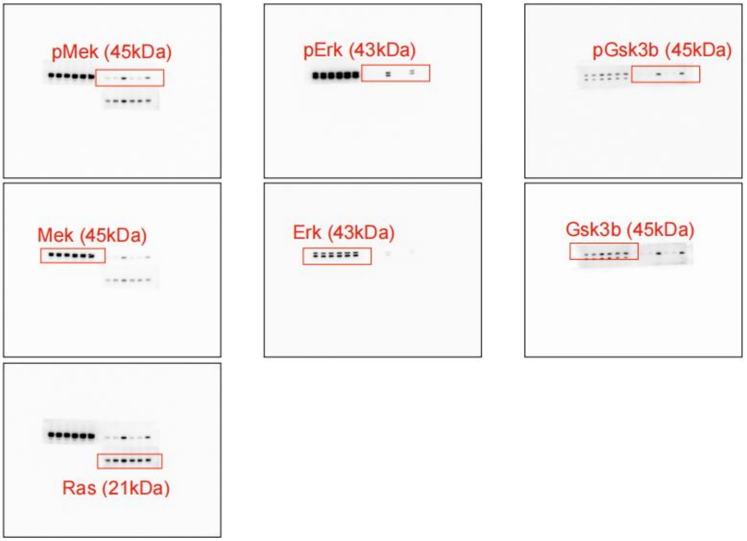

: from the same membrane

Mek | pMek
Ras

: from the same membrane

Erk | pErk

: from the same membrane

Gsk3b | pGsk3b

Figure 3A

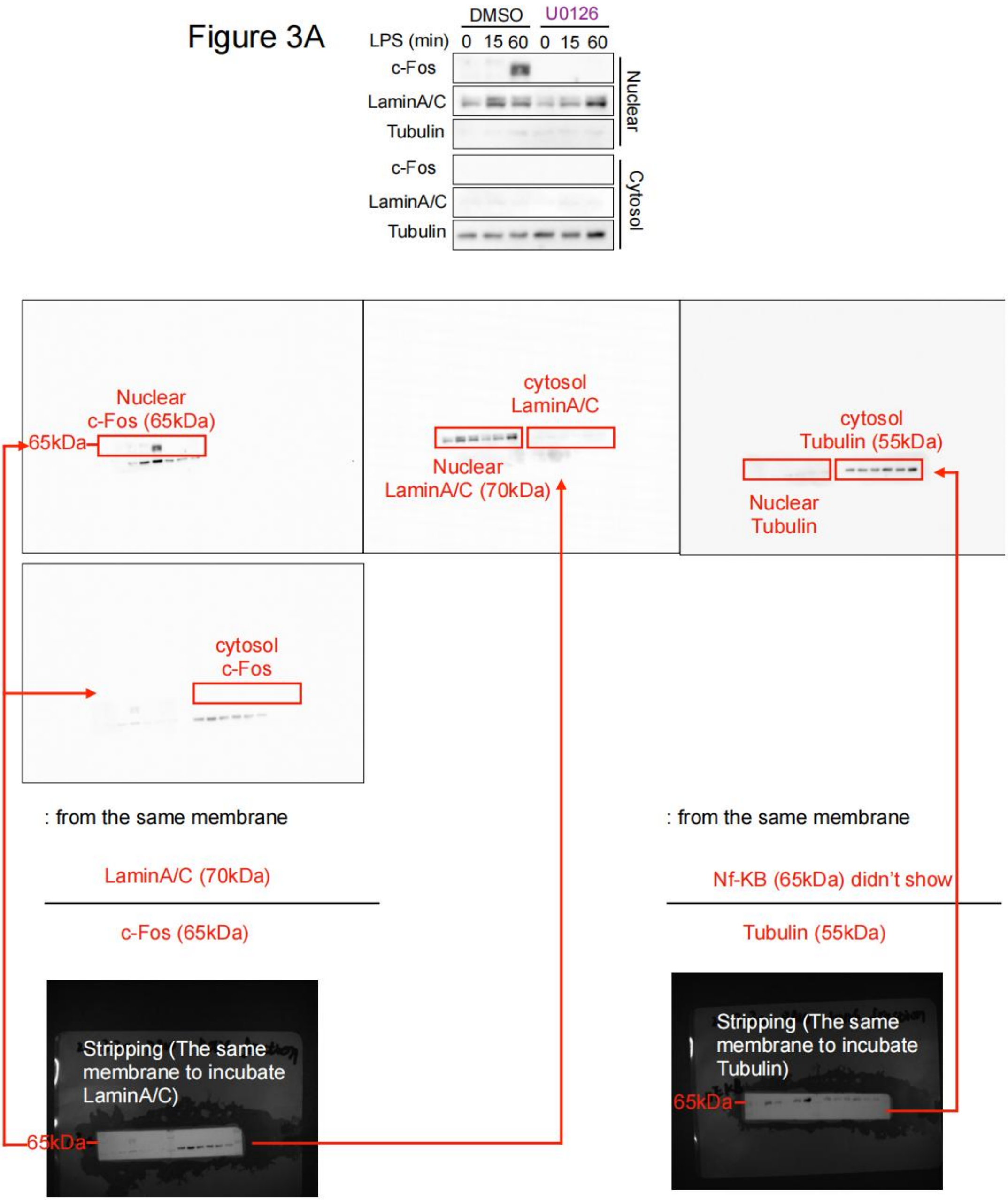
DMSO
U0126
LPS (min)
0 15 60 0 15 60
c-Fos
LaminA/C
Tubulin
c-Fos
LaminA/C
Tubulin
Nuclear
Cytosol
Nuclear
c-Fos (65kDa)
65kDa
cytosol
LaminA/C
Nuclear
LaminA/C (70kDa)
cytosol
Tubulin (55kDa)
Nuclear
Tubulin
cytosol
c-Fos
: from the same membrane
LaminA/C (70kDa)
c-Fos (65kDa)
: from the same membrane
Nf-KB (65kDa) didn't show
Tubulin (55kDa)
Stripping (The same membrane to incubate LaminA/C)
65kDa
Stripping (The same membrane to incubate Tubulin)
65kDa

Figure 3B

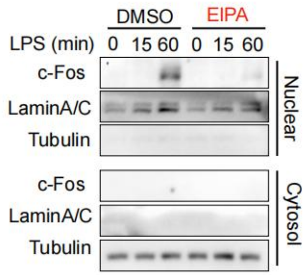
DMSO
EIPA
LPS (min)
0 15 60 0 15 60
c-Fos
LaminA/C
Tubulin
Nuclear
c-Fos
LaminA/C
Tubulin
Cytosol

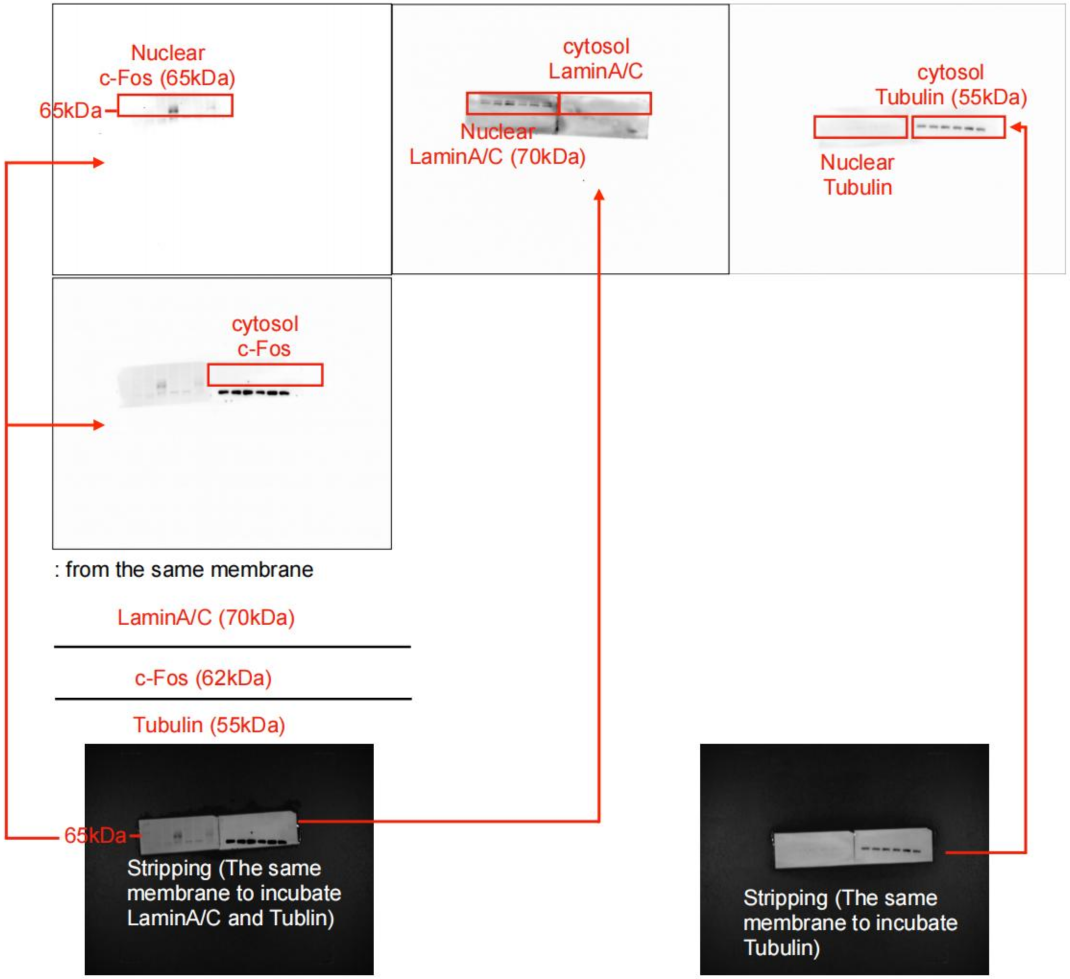
Nuclear
c-Fos (65kDa)
65kDa
cytosol
LaminA/C
Nuclear
LaminA/C (70kDa)
cytosol
Tubulin (55kDa)
Nuclear
Tubulin
cytosol
c-Fos
: from the same membrane
LaminA/C (70kDa)
c-Fos (62kDa)
Tubulin (55kDa)
65kDa
Stripping (The same membrane to incubate LaminA/C and Tublin)
Stripping (The same membrane to incubate Tubulin)

Figure 3C

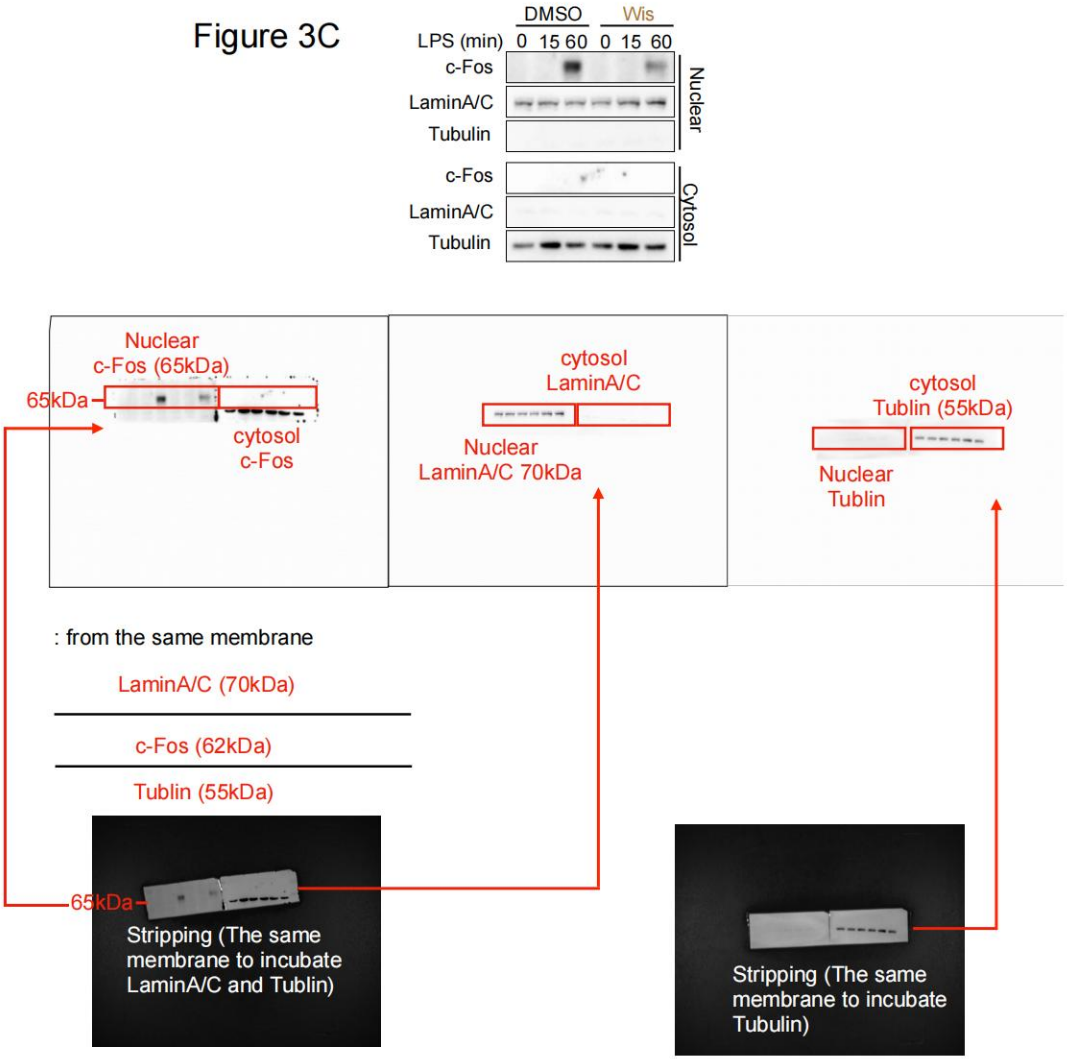

DMSO
Wis
LPS (min) 0 15 60 0 15 60
c-Fos
LaminA/C
Tubulin
Nuclear
c-Fos
LaminA/C
Tubulin
Cytosol
Nuclear c-Fos (65kDa)
65kDa
cytosol c-Fos
cytosol LaminA/C
Nuclear LaminA/C 70kDa
cytosol Tublin (55kDa)
Nuclear Tublin
: from the same membrane
LaminA/C (70kDa)
c-Fos (62kDa)
Tublin (55kDa)
65kDa
Stripping (The same membrane to incubate LaminA/C and Tublin)
Stripping (The same membrane to incubate Tubulin)

Figure 3D

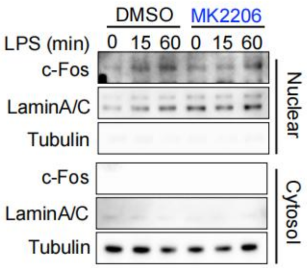
DMSO
MK2206
LPS (min)
0 15 60 0 15 60
c-Fos
LaminA/C
Tubulin
Nuclear
c-Fos
LaminA/C
Tubulin
Cytosol

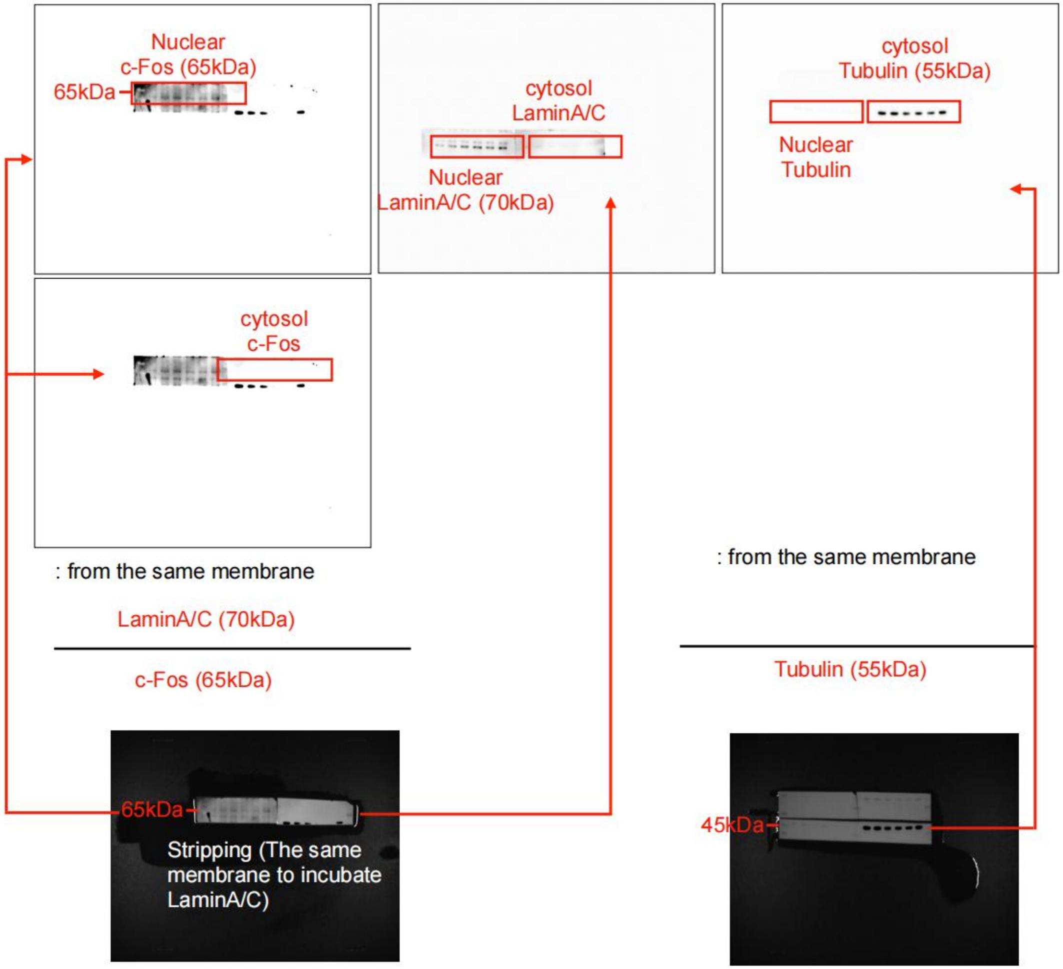
Nuclear
c-Fos (65kDa)
65kDa
cytosol
c-Fos
cytosol
LaminA/C
Nuclear
LaminA/C (70kDa)
cytosol
Tubulin (55kDa)
Nuclear
Tubulin
: from the same membrane
LaminA/C (70kDa)
c-Fos (65kDa)
65kDa
Stripping (The same membrane to incubate LaminA/C)
: from the same membrane
Tubulin (55kDa)
45kDa

S-Figure 3A-E

S-figure 3A

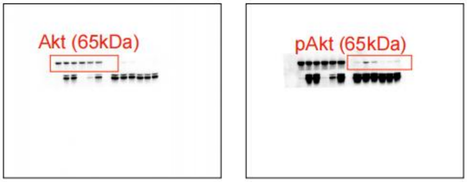

: from the same membrane

Akt (65kDa) | pAkt (65kDa)

S-figure 3B

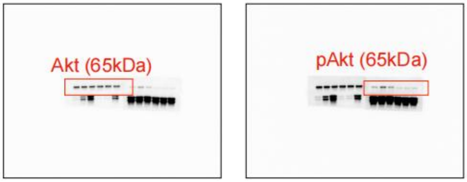

: from the same membrane

Akt (65kDa) | pAkt (65kDa)

S-figure 3C

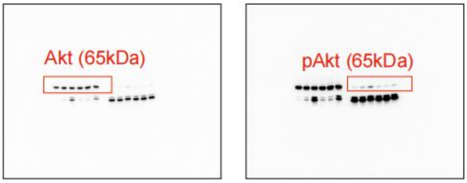

: from the same membrane

Akt (65kDa) | pAkt (65kDa)

S-figure 3D

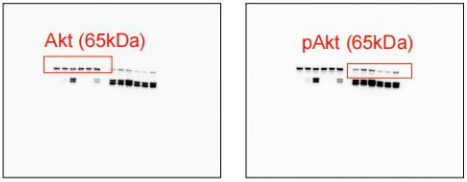

: from the same membrane

Akt (65kDa) | pAkt (65kDa)

S-figure 3E

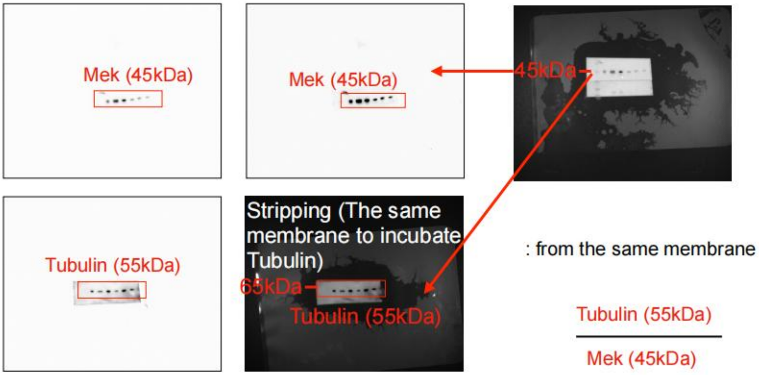

Supplement: Supplementary file 1 — Supplementary Materials [file csf_50_25008_1.pdf]
